# Embryonal Rhabdomyosarcoma of the Diaphragm in a Two-Year-Old Boy

**DOI:** 10.7759/cureus.103119

**Published:** 2026-02-06

**Authors:** Shuhei Sato, Takuya Kamio, Katsuyuki Tanaka, Taku Gomi, Masaharu Akiyama

**Affiliations:** 1 Pediatrics, The Jikei University School of Medicine, Tokyo, JPN; 2 Radiology, The Jikei University School of Medicine, Tokyo, JPN

**Keywords:** diaphragm, diaphragmatic crus, embryonal rhabdomyosarcoma, peritoneum, superior recess of omental bursa

## Abstract

Although primary tumors of the diaphragm are rare in children, rhabdomyosarcoma is the most common malignant tumor arising in the diaphragm. Rhabdomyosarcoma is a malignant tumor that arises from primary mesenchymal cells that differentiate into skeletal muscle. Here, we report on a two-year-old boy in whom embryonal rhabdomyosarcoma of the diaphragm developed and who presented with abdominal distension due to significant ascites. Contrast-enhanced computed tomography revealed two mass lesions: a lesion that had originated from the right diaphragmatic crus, part of the diaphragm, extending to the omental bursa, and another lesion in the left inguinal canal. Furthermore, massive ascites and diffuse peritoneal thickening suggested peritoneal dissemination. On 2-deoxy-2-(fluorine-18)-fluoro-D-glucose positron emission tomography combined with computed tomography, these lesions exhibited high uptake, suggesting malignant tumors. Pathological examination of biopsy specimens of the left inguinal tumor revealed embryonal rhabdomyosarcoma. According to the Intergroup Rhabdomyosarcoma Study IV risk classification system, the patient was categorized as high risk, meeting the criteria for embryonal-type rhabdomyosarcoma, stage 4, and group IV. The ARST0431 therapy was promptly started. Because tumor tissues disappeared on imaging after chemotherapy, surgical resection was omitted. No recurrence has been observed 16 months after the completion of treatment, including chemotherapy and radiotherapy. Even for a group IV rhabdomyosarcoma of the diaphragm, an embryonal type might improve the prognosis with multidisciplinary treatment. Long-term follow-up should carefully monitor not only recurrence but also side effects from the toxicity of total abdominal irradiation.

## Introduction

Primary tumors of the diaphragm are rare in children [1]. A study of 41 cases of pediatric diaphragmatic tumors reported from 1868 through 2005 revealed that the average age at diagnosis was 10 years, and that rhabdomyosarcoma was the most common malignant tumor [1]. Rhabdomyosarcoma is a malignant tumor that arises from primary mesenchymal cells that differentiate into skeletal muscle. The most common sites are the head and neck region (including the parameningeal and orbital areas), the genitourinary tract, and the extremities. However, rhabdomyosarcoma rarely develops in the diaphragm [2]. The diaphragm, a membranous muscle, is located at the boundary between the thoracic and abdominal cavities. Its structure consists of 3 layers: the diaphragmatic pleura, the muscular layer, and the diaphragmatic peritoneum [3]. The inferior surface of the diaphragm is covered by the transverse abdominal fascia and the peritoneum. Compared with rhabdomyosarcomas arising in common sites, primary tumors of the diaphragm can be more challenging to diagnose with imaging studies owing to the diaphragm's complex structure, adjacent organs, and intricately interwoven peritoneum. Furthermore, the deep location of the lesion can make even biopsy or resection difficult when the patient’s condition is poor. We report on a two-year-old boy with embryonal rhabdomyosarcoma of the diaphragm, which originated in the right diaphragmatic crus, spread to the omentum, and extensively spread to the peritoneum, and presented with abdominal distension due to significant ascites accumulation. This report emphasizes the specificity of the diaphragm as the site of origin, the critical importance of image-based diagnostic information, and the maintenance of remission of the primary tumor without surgery.

## Case presentation

A two-year-old boy who had had a cough and rhinorrhea for three days visited a nearby clinic, where an upper respiratory tract infection was diagnosed, and medication was prescribed. Although abdominal distension was noted, no further examination was performed. Because the upper respiratory infection showed no improvement two days later, the boy visited another clinic, from which he was referred to the Jikei University Katsushika Medical Center for further evaluation of the abdominal distension. An abdominal ultrasound examination performed there the following day revealed significant ascites, and a contrast-enhanced computed tomographic (CT) scan revealed tumor lesions near the gastroesophageal junction and in the left inguinal canal. Owing to the rapidly worsening abdominal distension and the possibility of a malignant tumor, the boy was referred to The Jikei University Hospital and admitted there the following day.

**Figure 1:**
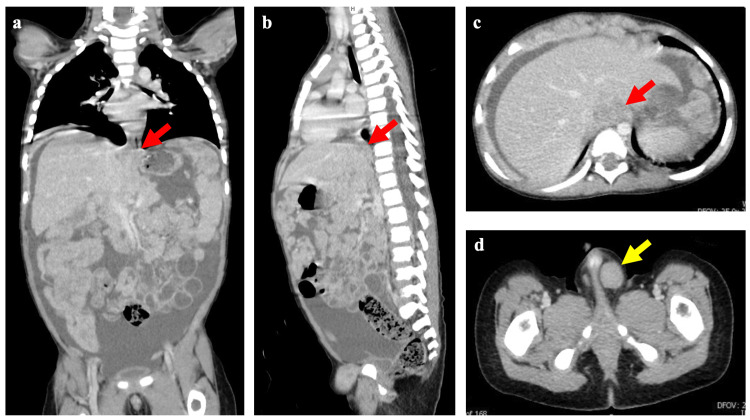
Contrast-enhanced chest and abdominal computed tomographic images. The red arrows indicate a tumorous lesion diagnosed as rhabdomyosarcoma on a coronal image (a), a sagittal image (b), and a horizontal image (c). The yellow arrow indicates a metastatic tumorous lesion of rhabdomyosarcoma on a horizontal image (d). The size of tumors originating from the diaphragm in panel (c) was 20.80 x 19.46 mm, while that of tumors in the left inguinal region in panel (d) was 12.30 x 13.80 mm.

The patient had been born via vaginal delivery at 40 weeks and two days of gestation. His medical and family history were not significant. Upon admission, his weight was 12.0 kg (-0.53 SD), and his height was 89.0 cm (-0.18 SD). A physical examination noted abdominal distension due to ascites and a firm, elastic mass with poor mobility in the left inguinal region. Blood examination showed reference ranges of tumor markers, such as carcinoembryonic antigen, α-fetoprotein, human chorionic gonadotropin, CA 19-9, and neuron-specific enolase. A contrast-enhanced CT examination of the abdomen revealed a mass lesion (20.80 x 19.46 mm) within the omental bursa and omental recess and a metastatic lesion (2.30 x 13.80 mm) extending from the left inguinal canal into the scrotum (Figure 1).

**Figure 2:**
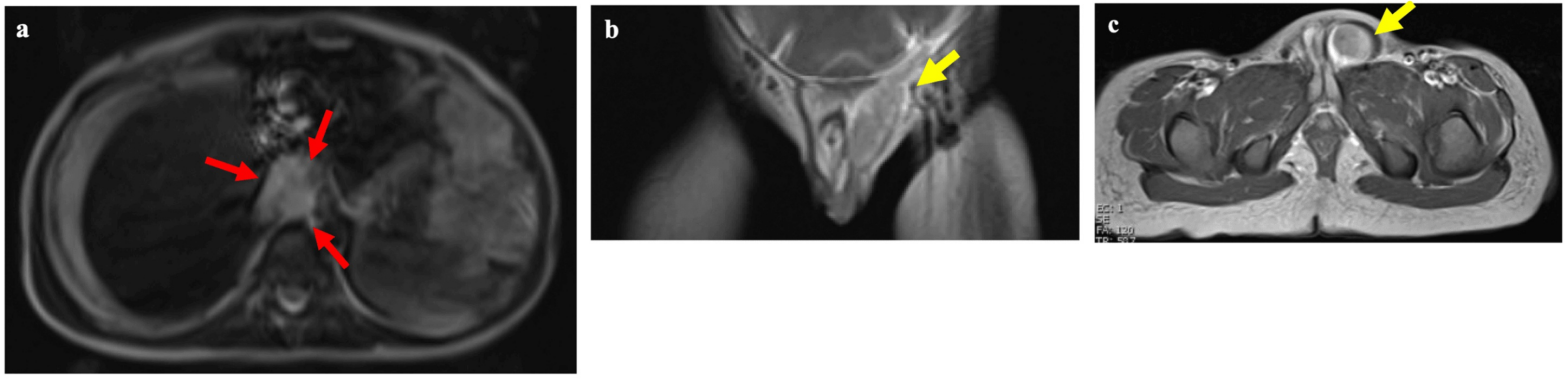
A contrast-enhanced magnetic resonance image of the chest and abdomen. The red arrows indicate the primary tumor lesion with mild hyperintensity on a horizontal T2-weighted image (a). The yellow arrow indicates a metastatic lesion extending from the left inguinal canal into the scrotum on a coronal contrast-enhanced T1-weighted image (b) and on a horizontal contrast-enhanced T1-weigted image (c). The size of tumors originating from the diaphragm in panel (a) was 18.19 x 24.26 mm, while that of tumors in the left inguinal region in panel (c) was 10.90 x 8.50 mm.

Massive ascites and diffuse peritoneal thickening were observed, suggesting peritoneal dissemination (Figure 1). Based on the enlargement of the left cardiodiaphragmatic angle lymph nodes, metastasis was suspected. A contrast-enhanced magnetic resonance image (MRI) of the chest and abdomen showed a primary tumor lesion with moderately high intensity on the T2-weighted image with diffusion restriction and contrast enhancement (Figure 2a). The lesion in the superior recess of the omental bursa was suspected to invade the posteriorly located right diaphragmatic crus, which showed low signal intensity on T2-weighted MRIs. Moreover, a metastatic lesion extended from the left inguinal canal into the scrotum on a sagittal contrast-enhanced T1-weighted image (Figure 2b) and on a horizontal contrast-enhanced T1-weighted image (Figure 2c). The size of tumors originating from the diaphragm was 18.19 x 24.26 mm (Figure 2a), and that of tumors in the left inguinal region was 10.90 x 8.50 mm (Figure 2c). The serosa was found to have invaded the left inguinal canal, leading us to conclude that tumor cells had spread via peritoneal dissemination and infiltrated the area (Figure 2b).

**Figure 3:**
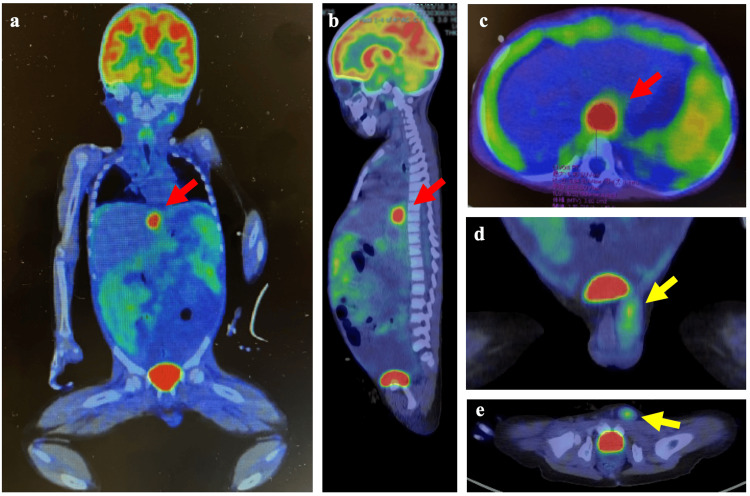
2-deoxy-2-(fluorine-18)-fluoro-D-glucose positron emission tomography/computed tomographic images. The red arrows indicate the primary tumor lesion on a coronal image (a), a sagittal image (b), and a horizontal image (c). The yellow arrow indicates a metastatic lesion extending from the left inguinal canal into the scrotum on a coronal image (d) and on a horizontal image (e). The volume of the neoplastic lesion originating from the diaphragm was 3.60 m³, and that of the neoplastic lesion in the left inguinal region was 0.41 m³.

A 2-deoxy-2-(fluorine-18)-fluoro-D-glucose (FDG) positron emission tomography (PET) scan combined with CT revealed high uptake (standardized uptake value [SUV] max 6.90) in an area consistent with the lesion, which extended from the superior recess of the omental bursa to the dorsal right diaphragmatic crus (Figure 3). Also observed were uptake in the peritoneum and a mass (SUV max 1.68) in the left inguinal region (Figure 3). The volume of the neoplastic lesion originating from the diaphragm was 3.60 m³, and that of the neoplastic lesion in the left inguinal region was 0.41 m³.

**Figure 4:**
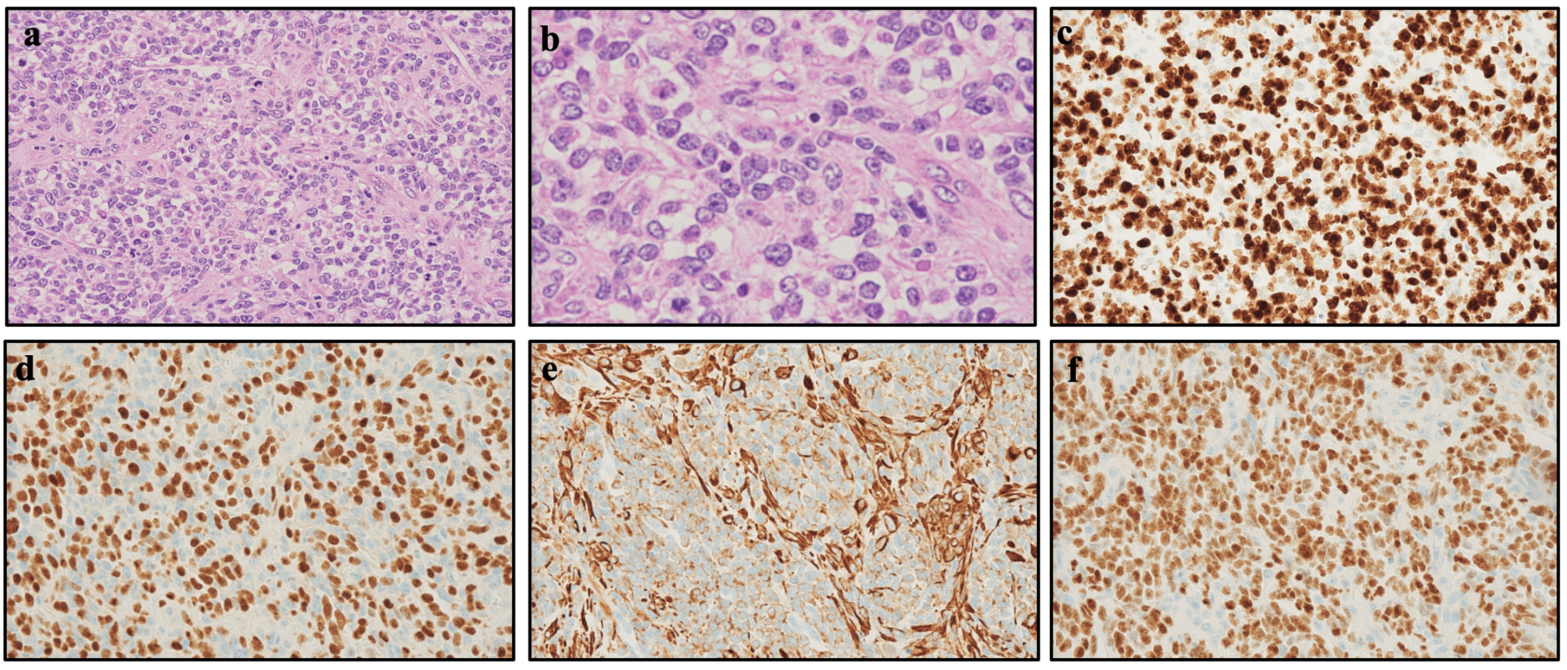
Pathological findings of the tumor biopsy sample obtained from the left inguinal lesion. (a) Hematoxylin-eosin staining (x400), (b) hematoxylin-eosin staining (x1000), (c) MIB-1 staining (x400), (d) myogenin staining (x400), (e) vimentin staining (x400), and (f) MyoD1 staining (x400).

A pathological examination was performed on tumor samples obtained via a biopsy of the inguinal lesion. Hematoxylin-eosin staining revealed tumor cells with a high nuclear and cytoplasmic ratio and nearly round nuclei that were densely proliferating (Figure 4). Nuclear mitosis was observed in 38 out of 10 high-power fields. Also noted were several rhabdomyoblasts with an eosinophilic cytoplasm. No anaplasia was observed. Immunohistochemical staining revealed an MIB1 index of 90% or higher. Positivity rates were high for desmin, myogenin, vimentin, and myoD1. Although small cells had proliferated densely, no distinct nests were evident. These pathological findings led to the diagnosis of high-density subtype embryonal rhabdomyosarcoma. The reverse transcription-polymerase chain reaction did not detect a PAX3/7::FOXO1 fusion gene.

Before the pathological diagnosis had been made, the patient received a course of chemotherapy consisting of vincristine, doxorubicin, and cyclophosphamide (VDC), which is expected to be effective for most solid tumors, because of suspected sarcomas such as rhabdomyosarcoma and Ewing sarcoma. According to the protocol risk classification of the Children’s Oncology Group’s ARST0431 study [4], the patient was considered to be at high risk, corresponding to rhabdomyosarcoma of embryonal type, stage 4, and group IV. Owing to this diagnosis, the ARST0431 therapy regimen was started [4]. The significant ascites was recognized to have gradually decreased with VDC chemotherapy alone and had resolved by the time ARST0431 therapy was started. Complete remission was confirmed in week six of reporting period 1 (RP1). The initial plan for radiotherapy was 24 Gy/16 fractions to the whole abdomen and pelvis, with 30.6 Gy/17 fractions to the local area (primary tumor site, diaphragm, left testis, and spermatic cord) and a 10.8 Gy/6 fraction boost to the tumor bed, resulting in a total dose of 41.4 Gy/23 fractions. However, one fraction of local irradiation was omitted, and 39.6 Gy in 22 fractions was administered when treatment with defibrotide sodium was started owing to liver damage caused by hepatic venoocclusive disease/sinusoidal obstruction syndrome. Furthermore, the chemotherapeutic agents were reduced in dosage as follows: cyclophosphamide by 50% at week 28; cyclophosphamide and actinomycin D by 50% at week 35; and actinomycin D by 25% at week 38. Considering VDC treatment had been administered before the pathological diagnosis, a VDC dose scheduled for RP3 of the ARST0431 protocol [5] was canceled. Sixteen months have passed since treatment was completed, and remission has been maintained.

## Discussion

To date, 19 cases of primary rhabdomyosarcoma of the diaphragm in patients 20 years or younger have been reported (Table 1) [1,2,5-13]. In the present patient, the reasons for determining the diaphragm lesion as the primary tumor and the left paratesticular lesion as a metastasis were based on the following three points: 1) The SUVmax values on PET-CT were 1.68 for the left paratesticular lesion and a significantly higher 6.90 for the diaphragm lesion. 2) The calculated volume of the uptake area on PET-CT was 0.41 cm³ for the left paratesticular lesion and a larger 3.6 cm³ for the diaphragm lesion. 3) The CT and MRI findings suggested serosal involvement in the left inguinal canal.

**Table 1:** Clinical characteristics of rhabdomyosarcoma of the diaphragm in patients 20 years or younger. B: biopsy; C: chemotherapy; D: debulk; F: female; M: male; R: radiotherapy; S: surgery

Case	Age (years)	Sex	Pathological subtype	Clinical group	Treatment	Outcome (+ years)	References
1	18	M	Embryonal	I	S + C	Survival + 1.3	Medeiros et al., 2002 [5]
2	17	F	Embryonal	III	B + C + R	Survival + 1.1	Raney et al., 2000 [2]
3	11	F	Embryonal	IV	D + C + R	Death	Raney et al., 2000 [2]
4	8	M	Embryonal	IV	B + C + R	Survival + 8.79	Raney et al., 2000 [2]
5	2	M	Embryonal	No data	S + C	Survival	Gupta et al., 1999 [6]
6	3	M	Embryonal	No data	S + C	Survival	Vade et al., 2000 [7]
7	3	M	Embryonal	No data	S + C + R	Survival + 1	Cada et al., 2006 [1]
8	4	F	Embryonal	No data	S + C	Survival + 2.6	Federici et al., 1986 [8]
9	2	M	Embryonal	IV	B + C + R	Survival + 1	Present case
10	2	F	Alveolar	III	B + C + R	Survival + 15.6	Raney et al., 200 [2]
11	9	F	Alveolar	III	B + C + R	Death	Raney et al., 2000 [2]
12	13	F	Alveolar	IV	B + C + R	Death	Raney et al., 2000 [2]
13	13	M	Alveolar	IV	D + C + R	Death	Raney et al., 2000 [2]
14	12	F	Alveolar	IV	B + C + R	Death	Raney et al., 2000 [2]
15	4	M	Alveolar	No data	No data	No data	Theunissen et al., 2004 [9]
16	4	M	Pleomorphic	I	S + C	Survival	Vano et al., 1988 [10]
17	20	F	Pleomorphic	IV	S + C + R	Death	Midorikawa et al., 1998 [11]
18	14	M	No data	No data	None	Death	Peery et al., 1939 [12]
19	12	M	No data	No data	No data	Death	Eustace et al., 1993 [13]

The symptoms of diaphragmatic tumors, including rhabdomyosarcoma, depend on the tumor’s size and location at the time of diagnosis [1]. Tumors on the left side of the body can cause digestive symptoms, such as anorexia, nausea, and vomiting, due to gastric compression [1]. Respiratory symptoms in reported cases have included cough, chest pain, and dyspnea [5,12]; abdominal symptoms have included appetite loss, dysphagia, abdominal distension, and abdominal pain [1,6,8,10]; and other symptoms have included fever and weight loss [9,12] (Table 1). A physical examination can reveal palpable masses in the chest or abdomen [1,6-8] (Table 1). In the present patient, the symptoms involved the upper airway and moderate abdominal distension, and the tumorous lesion was not noticed until the abdominal distension became more severe. Because symptoms are absent or nonspecific in most patients with diaphragmatic tumors, early diagnosis is difficult.

Rhabdomyosarcoma requires multidisciplinary therapy involving surgery, radiotherapy, and chemotherapy. A review of prognoses in 19 reported cases of primary diaphragmatic rhabdomyosarcoma, including the present case, reveals significant data insufficiency and a short follow-up period for embryonal tumors, making definitive conclusions difficult. Moreover, it should be noted that the present report is of a single case and that comparing it with reports of previous cases has methodological limitations. Following the pathological diagnosis of rhabdomyosarcoma of the diaphragm, the patient underwent ARST0431 therapy, the standard treatment for high-risk rhabdomyosarcoma. Because imaging studies, including FDG-PET/CT, had shown that the tumor lesions had disappeared, no additional surgery, including biopsy, was performed, and treatment was finally completed with chemotherapy and radiotherapy. Although our patient had a rhabdomyosarcoma of clinical group IV, its histological type was embryonal, and the absence of the PAX3/7::FOXO1 fusion gene, which has been reported as a favorable prognostic factor [14], suggested an improved prognosis. However, in cases for which remission is achieved with chemotherapy and radiotherapy, it remains unclear whether FDG-PET/CT is appropriate for evaluating treatment efficacy and whether omitting biopsy for histological confirmation of remission is appropriate [15]. Long-term follow-up is necessary to clarify these points.

## Conclusions

A combination of CT, MRI, and PET-CT imaging was useful for identifying the primary tumor site in the diaphragm. Owing to the patient’s significantly deteriorated general condition caused by severe ascites and pleural effusion, open biopsy or surgical resection of deep intra-abdominal lesions was difficult. However, remission has been maintained with chemotherapy and radiotherapy. Even for stage IV rhabdomyosarcoma of the diaphragm, an embryonal type might have improved the prognosis with multidisciplinary treatment. Long-term follow-up should carefully monitor not only recurrence but also side effects from the toxicity of total abdominal irradiation.
